# Supplementary material for: The Small RNA Universe of Capitella teleta
Source: Front Mol Biosci. 2022 Feb 25;9:802814. doi: 10.3389/fmolb.2022.802814 (PMC8915122; doi:10.3389/fmolb.2022.802814)
Supplement: Supplementary file 1 [file DataSheet1.ZIP › Supplement/known/CAPTEscaffold_122_9855.pdf]

Provisional ID	:	CAPTEscaffold_122_9855
Score total	:	7988.2
Score for star read(s)	:	3.9
Score for read counts	:	7978.9
Score for mfe	:	0.8
Score for randfold	:	1.6
Score for cons. seed	:	3
Total read count	:	15662
Mature read count	:	15605
Loop read count	:	0
Star read count	:	57

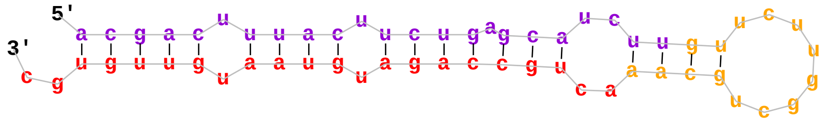

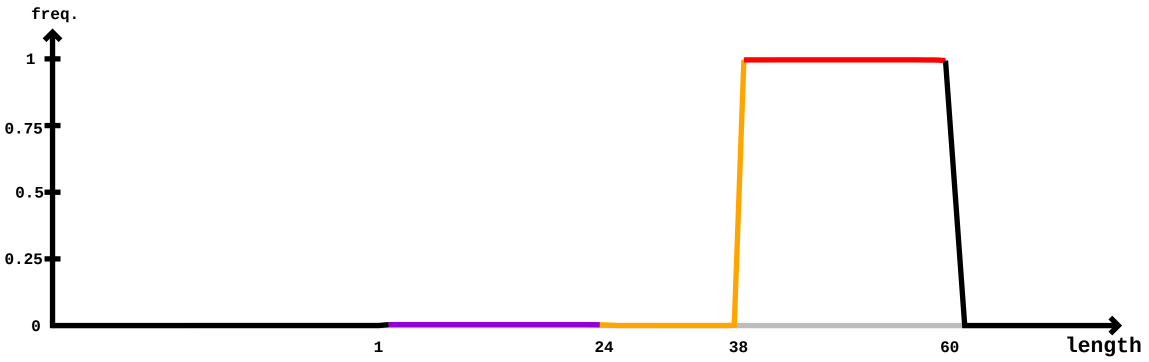

Star Mature

```
5'- cucaaccaugaccuuuguuugccagcgacaaucacgacuuuacuucugagcaucuuguucuuggcugcaaacugccagauguaaauguugugcacgaaguugguccagcc  -3'  obs
    cucaaccaugaccuuuguuugccagcgacaaucacgacuuuacuucugagcaucuuguucuuggcugcaaacugccagauguaaauguugugcacgaaguugguccagcc       exp
    cucaaccaugaccuuuguuugccagcgacaaucacgacuuuacuucugagcaucuuguucuuggcugcaaacugccagauguaaauguugugcacgaaguugguccagcc       known
    ................(((.((((((......((((((.((((.((((.(((..((((........))))..))))))).)))).))))))......))))))..))).   reads  mm  sample

    .............uuuguuugccagcgacaauc.............................................................................   1      0   seq
    .................................acgacuuuacuucugagcauc........................................................   2      0   seq
    .................................acgacuuuacuucugagcaucu.......................................................   8      0   seq
    .................................Gcgacuuuacuucugagcaucuu......................................................   1      1   seq
    .................................acgacuAuacuucugagcaucuu......................................................   1      1   seq
    .................................acgacuuuacuucugagcaucuu......................................................   43     0   seq
    .................................acgacuuuacuucugagcaucuuA.....................................................   1      1   seq
    .................................acgacuuuacuucugagcaucuuguucu.................................................   1      0   seq
    .....................................................................aacugccagauguaaauguugugc.................   4      0   seq
    .....................................................................Cacugccagauguaaauguugugc.................   13     1   seq
    ......................................................................acugccagauguaaauguug....................   6      0   seq
    ......................................................................acugccagauguaaauguugu...................   1      0   seq
    ......................................................................acugccagauguaaauguugug..................   36     0   seq
    ......................................................................acugccagauguaaGguugugc.................   1      1   seq
    ......................................................................acugccagauguaaauguuAugc.................   3      1   seq
    ......................................................................acugccagauguaaauguugCgc.................   4      1   seq
    ......................................................................acugccagGuguaaauguugugc.................   3      1   seq
    ......................................................................Ncugccagauguaaauguugugc.................   4      1   seq
    ......................................................................acugccagaCguaaauguugugc.................   3      1   seq
    ......................................................................Ccugccagauguaaauguugugc.................   3      1   seq
    ......................................................................acCgccagauguaaauguugugc.................   4      1   seq
    ......................................................................acugGcagauguaaauguugugc.................   1      1   seq
    ......................................................................acugccagaugAaauguugugc.................   11     1   seq
    ......................................................................acuCccagauguaaauguugugc.................   1      1   seq
    ......................................................................acugcAagauguaaauguugugc.................   5      1   seq
    ......................................................................acugccagauCuaaauguugugc.................   1      1   seq
    ......................................................................aAugccagauguaaauguugugc.................   3      1   seq
    ......................................................................acugccagauguaauguCgugc.................   2      1   seq
    ......................................................................acugccagauguGauguugugc.................   1      1   seq
    ......................................................................Gcugccagauguaaauguugugc.................   187    1   seq
    ......................................................................Ucugccagauguaaauguugugc.................   2      1   seq
```

Star **Mature**

cucaaccaugaccuuuguuugccagcgacaaucacgacuuuacuucugagcaucuuguucuuggcugcaaacugccagauguaauguugugcacgaaguugguccagcc			
....acugccagauguaauguuguAc.................	1	1	seq
....acugccagauguaaugCugugc.................	1	1	seq
....acuAccagauguaauguugugc.................	3	1	seq
....aUugccagauguaauguugugc.................	1	1	seq
....acugccagauguaauguugugA.................	10	1	seq
....acuUccagauguaauguugugc.................	1	1	seq
....acugccagaugCaauguugugc.................	1	1	seq
....acugccGgauguaauguugugc.................	4	1	seq
....acugccagauguaaCguugugc.................	3	1	seq
....acugccagauAuaauguugugc.................	2	1	seq
....acugccaCauguaauguugugc.................	1	1	seq
....acugccagauguaaAguugugc.................	7	1	seq
....acugccagauguaauguugGgc.................	1	1	seq
....acugccagaGguaauguugugc.................	1	1	seq
....acuNccagauguaauguugugc.................	1	1	seq
....acugccagauguaauguuCugc.................	2	1	seq
....acugUcagauguaauguugugc.................	2	1	seq
....acugccagauguaauguugugc.................	15080	0	seq
....acugccagauguaGuguugugc.................	1	1	seq
....acugccagauguaauAuugugc.................	3	1	seq
....acugccagauguaauguugAgc.................	6	1	seq
....acugccagauguaauguuguUc.................	1	1	seq
....acugccagauguaaugAugugc.................	8	1	seq
....acugccUgauguaauguugugc.................	2	1	seq
....acugccagauguaauguugugG.................	49	1	seq
....acugccagaAguaauguugugc.................	6	1	seq
....acugAcagauguaauguugugc.................	3	1	seq
....acGgccagauguaauguugugc.................	1	1	seq
....acugccagauUuaauguugugc.................	2	1	seq
....acugccagauguaauguAgugc.................	5	1	seq
....acugcUagauguaauguugugc.................	15	1	seq
....acugccaAauguaauguugugc.................	5	1	seq
....acugccagauguaauguugugU.................	6	1	seq
....acAgccagauguaauguugugc.................	47	1	seq
....acugccagUuguaauguugugc.................	1	1	seq
....acugccagauguaauguugugcC................	4	1	seq
....acugccagauguaauguugugcU................	16	1	seq
....cugccagauguaauguugugc.................	1	0	seq
....gccagauguaauguugugc.................	1	0	seq
....ccagauguaauguugugc.................	1	0	seq
